# Activated Memory Cytotoxic T-Lymphocytes and T-Cell Receptor Vβ Clonality Predict Treatment-Free Remission After Tyrosine Kinase Inhibitor Discontinuation in Chronic-Phase Chronic Myeloid Leukemia: A 1-Year Prospective Immuno-Monitoring Study

**DOI:** 10.3390/ijms27062713

**Published:** 2026-03-16

**Authors:** Tatsuro Jo, Yoshio Saburi, Taro Masunari, Kazuhiro Noguchi, Takahiro Sakai, Jun Taguchi, Eiichi Ohtsuka, Nobuo Sezaki, Ritsuko Kubota-Koketsu, Toru Kiguchi

**Affiliations:** 1 Department of Hematology, Japanese Red Cross Nagasaki Genbaku Hospital, Mori-machi 3-15, Nagasaki City 852-8511, Nagasaki, Japan; 2 Department of Hematology, Oita Prefectural Hospital, Toyo 2-8-1, Oita City 870-8511, Oita, Japan; 3 Department of Infectious Disease, Chugoku Central Hospital, Miyukicho Oazakamiiwanari 148-13, Fukuyama City 720-0001, Hiroshima, Japan; 4 Department of Clinical Laboratory, Japanese Red Cross Nagasaki Genbaku Hospital, Mori-machi 3-15, Nagasaki City 852-8511, Nagasaki, Japan; 5 Department of Hematology, Chugoku Central Hospital, Miyukicho Oazakamiiwanari 148-13, Fukuyama City 720-0001, Hiroshima, Japan; 6 Specimen Analysis Team, Human Resource Development Division, Center for Infectious Disease Education and Research, The University of Osaka, Yamadaoka 2-2, Suita City 565-0871, Osaka, Japan; 7 Division of Diabetes, Endocrinology and Hematology, Saitama Medical Center, Dokkyo Medical University, Minamikoshigaya 2-1-50, Koshigaya City 343-8555, Saitama, Japan

**Keywords:** chronic myeloid leukemia, cytotoxic T lymphocyte, treatment-free remission, tyrosine kinase inhibitor, flow cytometry, T-cell receptor repertoire, deep molecular response

## Abstract

We prospectively evaluated whether cytotoxic T-lymphocyte (CTL) activation and T-cell receptor (TCR) Vβ clonality predict treatment-free remission (TFR) after tyrosine kinase inhibitor (TKI) cessation in chronic-phase chronic myeloid leukemia (CML). Forty-five patients with sustained deep molecular response (DMR) were enrolled (On-TKI, *n* = 38; Off-TKI, *n* = 7) and underwent one-year immuno-monitoring from consent. The primary endpoint was 12-month TFR, defined as retention of MR4. Overall, 32/45 patients (71%) maintained TFR at 12 months. Longer TKI exposure and stable DMR were associated with TFR; notably, patients fulfilling “≥7 years of TKI plus ≥1 year of DMR” and exhibiting CTL activation features—CD8 > CD4, memory > effector, and/or highly activated CTL clones on TCR Vβ repertoire—showed the highest likelihood of durable TFR. By contrast, NK cells, effector Tregs, and G-/M-MDSCs did not discriminate TFR status in this cohort. Although antigen specificity against CML stem cells was not directly tested, the memory-dominant CTL phenotype is consistent with immune control after antigen reduction. These findings suggest that a simple, clinically accessible strategy based on flow cytometric CTL profiling and TCR Vβ clonality may help inform TKI discontinuation decisions in CML. External validation is warranted to confirm transportability and refine clinical thresholds.

## 1. Introduction

Several clinical factors have been associated with treatment-free remission (TFR) in chronic-phase chronic myeloid leukemia (CP–CML), including low Sokal and EUTOS Long-Term Survival (ELTS) scores, ≥5 years of tyrosine kinase inhibitor (TKI) therapy, and sustained deep molecular response (DMR) for at least 2 years [1]. However, even among patients who meet these criteria, approximately 38–61% fail to achieve durable TFR [2,3,4,5,6,7,8]. This indicates that TKIs are highly effective at inducing DMR; however, their ability to eradicate leukemic stem cells (LSCs) and sustain TFR remains limited.

Conversely, curative outcomes in CML have been observed with allogeneic hematopoietic stem cell transplantation (allo-HSCT) and interferon-alpha therapy [9,10]. In successful allo-HSCT, cytotoxic T lymphocytes (CTLs) exhibit a graft-versus-leukemia effect primarily by recognizing minor-histocompatibility antigens presented by host antigen-presenting cells [11,12]. Further, donor leukocyte infusion (DLI) induces molecular remission in case of post-HSCT relapse, highlighting the importance of cellular immunity targeting residual leukemic clones [13]. These findings suggest that adaptive immunity, particularly CTLs, may contribute to the immune control of residual CML stem cells.

Recent studies have revealed fluctuating molecular response (MR) depth levels in patients with TFR, indicating an equilibrium between LSC re-expansion and immune surveillance [14,15,16]. In this context, immune profiling has become increasingly relevant. For instance, patients with a lower CD4/CD8 ratio are more likely to sustain TFR [17], whereas increased programmed cell death protein 1 expression on CD8^+^ T-cells is associated with molecular relapse [18]. Innate immune components, such as natural killer (NK) cells, have been associated with successful TFR in some cohorts [19,20,21]. However, the elimination of malignant clones frequently requires sustained activity from effector and memory CTLs [22,23].

We previously reported that a patient with CML who maintained long-term DMR after dasatinib discontinuation demonstrated memory CTLs with clonal T-cell receptor (TCR) expansion [24]. Further, we revealed that CTL clonal expansion occurs more rapidly and robustly in patients treated with dasatinib than with other TKIs [25]. Moreover, effector CTL clonality was observed in a patient with positive T315I refractory to both dasatinib and ponatinib who remained in CP without clinical symptoms [26]. These observations indicate the involvement of CTL clonal activation against LSCs in maintaining disease control.

This prospective observational multicenter study aimed to clarify the association between TFR and CTL clonal dynamics in patients with CP–CML who had achieved long-term DMR. By longitudinally analyzing MR and immunophenotypic profiles, we aimed to identify predictive markers of TFR according to tumor immunity, focusing on CTL activity and memory–effector composition.

## 2. Results

### 2.1. Patient Characteristics

The clinical characteristics of the patients at baseline are summarized in Table 1. Of the 45 patients, 38 had maintained DMR for ≥1 year with TKI treatment (On-TKI group) and seven had maintained DMR for ≥1 year after TKI discontinuation (Off-TKI group). The median duration of TKI treatment was 7.65 years (1.5–17.4) in the On-TKI group and 10.0 years (3.3–18.3) in the Off-TKI group. In the Off-TKI group, the time from TKI discontinuation to study entry ranged from 1.1 to 7.1 years. At diagnosis, the Sokal, Hasford, and ELTS prognostic scores classed nine (20%), four (8.9%), and zero (0%) patients as high-risk, respectively.

### 2.2. Loss or Maintenance of MR

**Figure 1:**
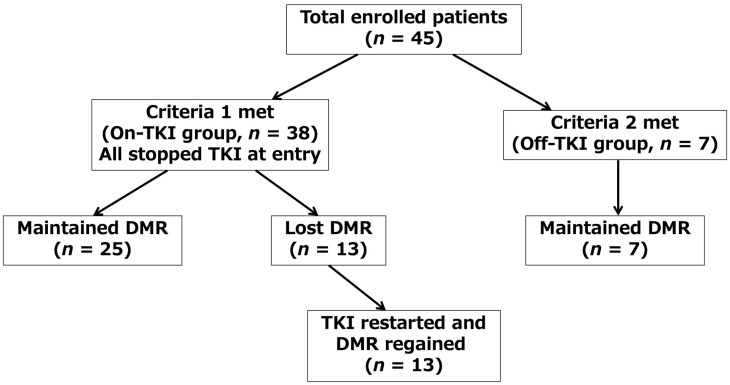
Consort diagram. The On-TKI-group included patients who had maintained DMR for >1 year with TKI treatment. The Off-TKI group included patients who had maintained DMR for >1 year after TKI discontinuation. DMR, deep molecular response; TKI, tyrosine kinase inhibitor.

The loss or maintenance of DMR in each group is shown in Table 2. In the On-TKI group, 25 (65.8%) patients, and in the Off-TKI group, all seven patients (100%), maintained their DMR throughout the study period. The median TKI treatment duration among patients who maintained DMR (achieved TFR) was significantly longer than that of patients who lost their DMR (*p* = 0.005). These data support previous reports suggesting that a longer TKI treatment duration is important for achieving TFR [1,2,3,4,5,6,7,8]. The 13 patients who lost their DMR resumed TKI treatment and all successfully regained DMR. These outcomes are illustrated in the consort diagram (Figure 1). Kinase domain mutation analysis was not performed in these 13 patients.

### 2.3. Rates of CD4, CD8, and CTL Clonality

CD4/CD8 T-cell ratios and CTL clonality in patients who lost their DMR, despite >10 years of TKI treatment, are summarized in Table 3. Among the 13 patients who lost their DMR, four had been treated with TKIs for >10 years. Three of those four had MR5 at the time of TKI discontinuation, and one had MR4.5. In all four, the CD4/CD8 ratio was >1 (CD4 > CD8), and the total CTL ratio was >1 (effector CTLs > memory CTLs). Activated effector and/or memory CTL clones were observed in patients LD-4, LD-7, and LD-13, whereas a highly activated effector CTL clone was detected in patient LD-11. No highly activated memory CTL clones were observed. These results indicate that the following immunological conditions may be important for achieving TFR: (1) CD8 predominance over CD4 (CD8 > CD4) in the T lymphocyte ratio, (2) memory CTLs predominating over effector CTLs (memory CTLs > effector CTLs) in the total CTL ratio, and (3) highly activated memory CTL clones.

Among 9 patients who had received <10 years of TKI treatment and lost DMR (Supplemental Table S1), 4 had CD8 > CD4, 5 had memory CTLs > effector CTLs, and 1 had a highly activated memory CTL clone—this patient had the shortest treatment duration (1.5 years). These results indicate that the above three immune factors may contribute to TFR, but are insufficient without adequate TKI treatment duration.

**Figure 2:**
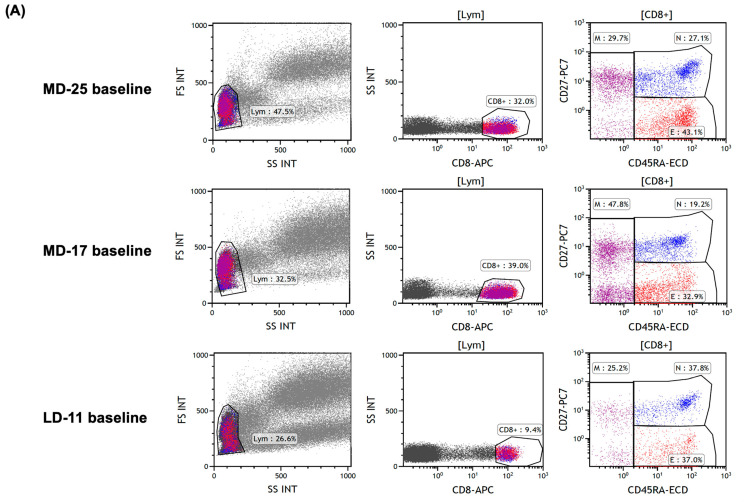

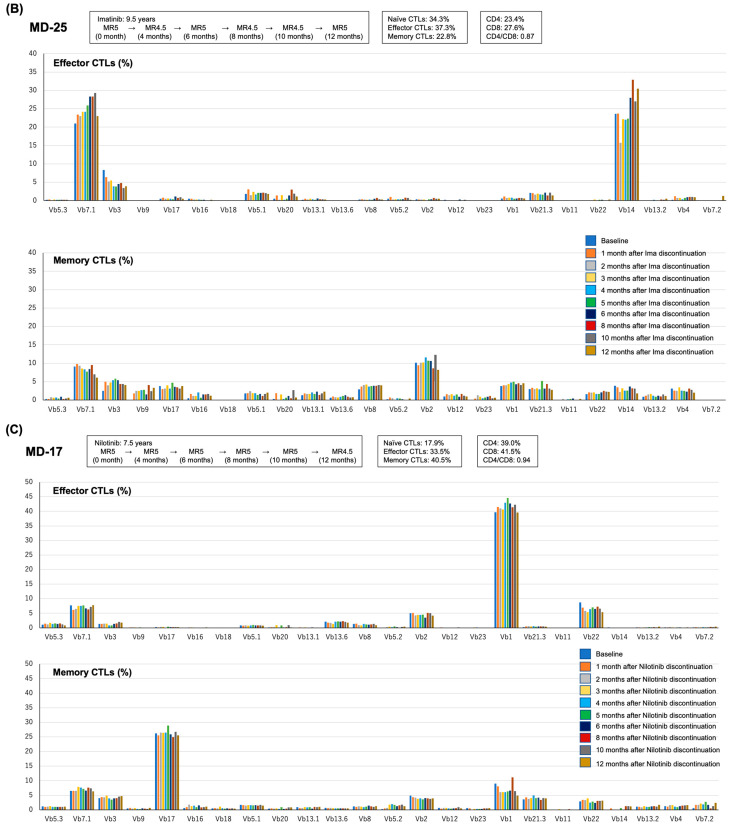

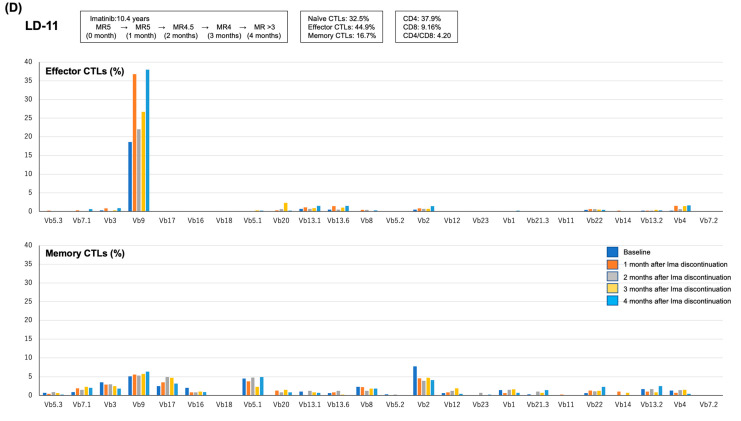
Representative baseline gating strategy and longitudinal changes in molecular response and CTL/TCR Vβ repertoire after TKI discontinuation in CP–CML. (**A**) Baseline flow cytometric plots from three representative patients displayed side by side: MD-25, who maintained TFR despite fluctuations in MR depth; MD-17, who maintained TFR for 12 months after TKI discontinuation; and LD-11, who lost DMR at 4 months after discontinuation. For each patient, the sequential gating strategy is shown as FSC/SSC lymphocyte gating, identification of CD8^+^ T-cells, and subdivision into naïve, effector, and memory CTL subsets using CD27 and CD45RA. (**B**) Longitudinal MR and TCR Vβ repertoire patterns in patient MD-25 from baseline to 12 months after imatinib discontinuation. MR depth fluctuations and persistence of highly activated effector CTL clones (Vβ7.1 and Vβ14) were observed. The patient had received imatinib for 9.5 years before discontinuation. (**C**) Longitudinal MR and TCR Vβ repertoire patterns in patient MD-17 from baseline to 12 months after nilotinib discontinuation. Highly activated effector and memory CTL clones were observed (Vβ1 and Vβ17, respectively). The patient had received nilotinib for 7.5 years before discontinuation. (**D**) Longitudinal MR and TCR Vβ repertoire patterns in patient LD-11 from baseline to 4 months after imatinib discontinuation, at which time DMR was lost. A highly activated effector CTL clone was observed (Vβ9), whereas no highly activated memory CTL clone was detected. The patient had received imatinib for 10.4 years before discontinuation. TCR Vβ analysis was performed on CD8^+^ T-cells (CD8/CD27/CD45RA plus the TCR repertoire kit). Naïve, effector, and memory CTL percentages are expressed as proportions of total CD8^+^ T-cells. CD4 and CD8 values indicate percentages among lymphocytes. In panels (**B**–**D**), the values shown for naïve/effector/memory CTLs and CD4/CD8 metrics represent mean values across the observation period for each patient (baseline to 12 months for MD-25 and MD-17; baseline to 4 months for LD-11) and are not baseline values. Abbreviations: CP-CML, chronic-phase chronic myeloid leukemia; CTL, cytotoxic T lymphocyte; DMR, deep molecular response; FS INT, forward scatter intensity; Ima, imatinib; Lym, lymphocyte; MR, molecular response; SS INT, side scatter intensity; TCR, T-cell receptor; TFR, treatment-free remission; TKI, tyrosine kinase inhibitor; Vb, TCR Vβ family.

As patient LD-8 lost DMR after 6.1 years of TKI treatment, we estimated that ≥7 years of TKI therapy may be necessary. We then analyzed 21 patients with TKI ≥7 years and successful TFR (Supplemental Table S2). Of these, four had both CD8 > CD4 and highly activated effector CTL clones; one also had a highly activated memory CTL clone. Six patients had memory CTLs > effector CTLs. Of these patients, 4 had highly activated effector CTL clones, and 1 had both effector and memory highly activated clones. In total, 12 out of 21 patients had highly activated effector CTL clones. By adding LD-11 (who received >10 years of TKI and lost DMR) to this group, we calculated that 12/13 (92.3%) patients with (a) ≥7 years of TKI treatment, (b) ≥1 year of DMR, and (c) highly activated effector CTL clones achieved TFR. Figure 2 presents representative flow cytometric plots and longitudinal immuno-monitoring data illustrating the relationship between MR dynamics and CTL phenotypes/TCR Vβ repertoire patterns after TKI discontinuation. Figure 2A shows baseline gating plots from three representative patients displayed side by side: MD-25, who maintained TFR despite fluctuations in MR depth; MD-17, who maintained TFR for 12 months; and LD-11, who lost DMR at 4 months after discontinuation. Longitudinal changes in MR and the TCR Vβ repertoire within effector and memory CTL subsets are shown for each patient in the subsequent panels (MD-25, Figure 2B; MD-17, Figure 2C; LD-11, Figure 2D). Among these representative cases, MD-17 fulfilled the proposed ‘immunologically safe discontinuation’ profile (CD8 > CD4 and memory CTLs predominating over effector CTLs) and showed highly activated effector and memory CTL clones (Vβ1 and Vβ17, respectively) during follow-up, whereas LD-11 showed CD4 > CD8 with effector CTLs predominating over memory CTLs and lacked a highly activated memory CTL clone (with a highly activated effector CTL clone, Vβ9), and subsequently lost DMR. For clarity, the naïve/effector/memory CTL proportions and CD4/CD8 metrics displayed in the upper panels of Figure 2B–D represent mean values across the observation period (baseline to 12 months for MD-25 and MD-17; baseline to 4 months for LD-11), not baseline values.

### 2.4. Conditions Required for Safe TKI Discontinuation

Table 4 shows the three immune-based conditions predictive of safe TKI discontinuation in CP–CML patients. These were applied to the seven Off-TKI group patients. Five had TKI ≥7 years; three met safety condition 1, and two met safety condition 2. Among the two patients with <7 years of TKI (3.3 and 5.2 years), one had a highly activated memory CTL clone, and one had both CD8 > CD4 and memory CTLs > effector CTLs (Supplemental Table S3). Thus, safety conditions 1 and 2 may serve as useful immunological indicators for safe TKI discontinuation.

In an exploratory analysis restricted to patients with ≥7 years of TKI therapy, we compared immunological features between those who maintained DMR (*n* = 21) and those who lost DMR after discontinuation (*n* = 4) (Table 5). The proportion of patients with CD8 > CD4 T-cell ratio differed between groups (16/21 vs. 0/4; Fisher’s exact test), whereas the other candidate conditions—predominance of total memory over effector CTLs (7/21 vs. 0/4), presence of a highly activated memory CTL clone (3/21 vs. 0/4), and presence of a highly activated effector CTL clone (10/21 vs. 1/4)—did not reach statistical significance in this limited dataset. Given the small number of events, these comparisons should be interpreted as hypothesis-generating.

### 2.5. Other Biological Markers

**Figure 3:**
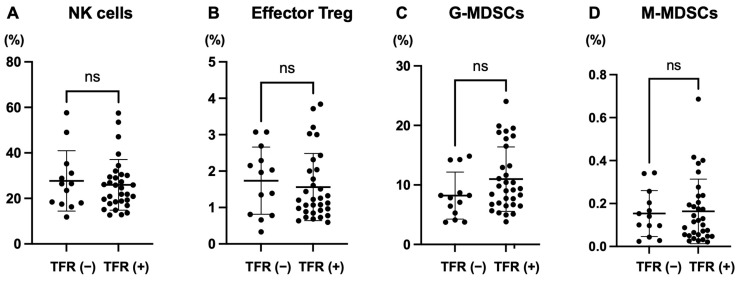
Comparison of immune markers other than CTL between CP–CML patients who did and did not achieve TFR during the observation period. (**A**) NK cell ratio; (**B**) Effector Treg ratio; (**C**) G-MDSC frequency; (**D**) M-MDSC frequency in the peripheral blood. NK cells were defined as CD3^−^CD16^+^CD56^+^. Effector Tregs were defined as CD4^+^CD45RA^−^FoxP3^++^. G-MDSCs were defined as HLA-DR^−^CD11b^+^CD33^+^CD14^−^. M-MDSCs were defined as HLA-DR^−^CD11b^+^CD33^+^CD14^+^. G-MDSC ratio and M-MDSC ratio were defined as the percentage of G-MDSCs or M-MDSCs among total nucleated cells in whole blood, respectively. Representative flow cytometric gating plots for T-cell subsets, NK cells, effector Tregs, G-MDSCs, and M-MDSCs are provided in Supplemental Figure S1. TFR (−): Patients who lost DMR during the observation period; TFR (+): Patients who were in TFR throughout the observation period. CP–CML, chronic-phase chronic myeloid leukemia; CTLs, cytotoxic T lymphocytes; DMR, deep molecular response; MDSCs, myeloid-derived suppressor cells; NK, natural killer; TFR, treatment-free remission; Treg, regulatory T-cell.

No significant differences were found in the NK cells, effector Tregs, or G-/M-MDSCs between patients who achieved and those who did not achieve TFR (Figure 3). Representative gating plots for T-cell subsets, NK cells, effector Tregs, G-MDSCs, and M-MDSCs are provided in Supplemental Figure S1.

### 2.6. Exploratory Analysis of Prior Dasatinib Exposure

To address whether prior TKI type may influence CTL subset distribution, we compared patients with a history of dasatinib treatment (*n* = 21) versus those without (*n* = 24). The mean proportion of total memory CTLs during the observation period was higher in the dasatinib-exposed group (unpaired *t*-test, *p* = 0.0108), whereas the mean proportion of total effector CTLs did not differ between groups (*p* = 0.3650) (Supplemental Figure S2).

## 3. Discussion

This observational study explored the role of tumor immunity in the achievement of TFR among patients with CP–CML. We observed that CTL activation features (particularly a higher proportion of memory CTLs), a sufficiently long TKI treatment period (≥7 years), and maintenance of DMR for ≥1 year were associated with TFR in this cohort (Table 4).

The clinical importance of CTL activation has been demonstrated by the beneficial effects of allo-HSCT, interferon-alpha, and DLI, and studies of CML-specific tumor antigens [9,10,11,12,13,14,15,16,17,18,19,20,21]. Thus, our results align with existing evidence. The decision to discontinue TKI treatment must be considered carefully by CML patients and their physicians. While numerous biomarkers for TKI discontinuation have been proposed, none have yet been definitively established [27,28,29,30,31,32,33,34]. The present study suggests that a simple flow cytometry test may provide a practical approach to risk stratification when considering TKI discontinuation. To provide statistical support for these candidate immunological conditions, we performed univariate comparisons among patients treated with TKIs for ≥7 years, contrasting those who maintained DMR (*n* = 21) with those who experienced DMR loss after discontinuation (*n* = 4) (Table 5). Among the four candidate conditions, only a CD8 > CD4 T-cell ratio was significantly associated with sustained DMR (Fisher’s exact test, *p* = 0.010). The remaining conditions (predominance of memory over effector CTLs and the presence of highly activated effector or memory CTL clones) did not reach statistical significance, which is likely attributable to limited power and zero-cell counts in this small cohort. Therefore, these results should be interpreted as supportive but exploratory, reinforcing CD8/CD4 balance as a robust immunological correlate while underscoring the need for validation in larger datasets.

The genetic biomarkers considered for TFR prediction in CP–CML patients include quantification of *bcr-abl1* transcripts, telomere length, and expression level of *ABCG2* [27,28,30,31]. However, these tests lack the speed, ease, and efficiency needed in clinical practice. An immunological study on TFR in CP–CML found that higher number of NK cells were associated with superior relapse-free survival rates [32,34]. Another report found significantly higher TCRγδ CD3^+^CD8^+^ T lymphocyte ratios in patients who achieved TFR [33]. Additionally, the numbers of certain immunosuppressive cells, including plasmacytoid dendritic cells, Tregs, and M-MDSCs were significantly lower in patients who achieve TFR [18,34]. These results support our findings that indicate the importance of tumor immunity in TFR acquisition.

We identified no significant differences in the NK cell, effector Treg, and G-MDSC, or M-MDSC ratios between patients who did and did not achieve TFR (Figure 3). This observation contrasts with previous studies that reported a correlation between elevated NK cell counts and successful TFR outcomes [17,34]; however, this discrepancy should not be interpreted as a refutation of the involvement of innate immunity in TFR. NK cells are well-established contributors to antitumor immunity, particularly in the early phases of leukemic control. However, robust and sustained tumor clearance, especially in the context of minimal residual disease, typically requires engagement of the adaptive immune system, particularly antigen-specific CTLs [22,23]. In addition to NK cells and Tregs, MDSCs are key regulators of antitumor immunity and have been potential correlates of TFR in CML in a prior study [34]. In our cohort, however, we did not observe significant differences in either G- or M-MDSC frequencies between patients who maintained TFR and those who experienced molecular relapse (Figure 3). This negative finding should not be interpreted as evidence against a biological role for MDSCs in TFR; rather, it may reflect differences in cohort composition, sampling times, and methodological definitions used to quantify MDSCs across studies. Notably, in this study MDSCs were expressed as frequencies among total nucleated cells in whole blood to provide a normalized metric for longitudinal monitoring. We acknowledge that absolute counts, which require volumetric acquisition or counting beads, may provide additional granularity, particularly when leukocyte counts fluctuate, and should be incorporated in future studies. We identified no dynamic changes in NK cells, Tregs, or MDSCs during the one-year immuno-monitoring period in our cohort. This likely reflects the distinct immunological window and cellular hierarchy governing late-stage disease control. Our results indicate the predictive value of effector and memory CTL activation and clonality for TFR acquisition. In an exploratory comparison, patients with prior exposure to dasatinib showed a higher mean proportion of memory CTLs over the monitoring period than those without dasatinib exposure, while effector CTL proportions were not significantly different (Supplemental Figure S2). This observation is consistent with our previous reports suggesting that dasatinib may modulate T-cell subsets; however, this study was not designed to compare TKIs, and the analysis is susceptible to confounding (e.g., treatment duration, patient selection, and timing of measurements). Therefore, the finding should be interpreted as hypothesis-generating and warrants validation in larger, TKI-stratified cohorts.

The predominance of memory over effector CTLs among TFR achievers may indicate a state of reduced burden of CML stem cells. TKIs are known to effectively suppress proliferating leukemic cells but fail to eradicate quiescent CML stem cells [35]; thus, long-term immune surveillance is crucial for maintaining remission after TKI discontinuation. As in other tumor systems, including chronic viral infection and solid cancers, contraction of the effector CTL pool and maintenance of a memory subset capable of long-term immune surveillance frequently follow effective antigen clearance [19,22,23]. Hence, memory CTLs, which can rapidly reactivate upon minimal antigen reappearance, may contribute to long-term immune surveillance. The presence of highly activated memory CTL clones in patients who maintained TFR is consistent with functional anti-CML immunity. Although our study did not directly quantify LSCs, the observed association between CTL activation and sustained DMR, together with MR depth fluctuations in some patients (Figure 2), supports the hypothesis that CTL may counteract transient expansions of minimal residual disease. Thus, immunophenotypic profiling of memory CTLs may serve as an accessible surrogate marker of immune-mediated disease control and a candidate marker for durable TFR readiness. Our flow cytometry-based model may provide a clinically implementable framework to support decision-making, while requiring validation in larger independent cohorts.

However, this study had several limitations. These included its small sample size. We hope that the index derived from this study will be tested in clinical practice to confirm its utility. Another limitation was that the TCR repertoire kit used may not have included all *TCR Vβ* genes. Therefore, we cannot rule out the possibility that patients who were found to have no highly activated effector or memory CTL clones may have had activated CTLs with *TCR Vβ* genes that were not tested. In such cases, the indicators “CD8 > CD4 in T lymphocyte ratio” and “memory CTLs > effector CTLs in total CTLs” would be helpful. In this study, we did not aim to directly identify the specificity of highly activated CTLs observed in patients who achieve TFR to CML stem cells. However, the association observed between TFR success and CTL activation provides indirect support for their functional relevance. While we observed that the activation status and clonality within phenotypically defined CD8^+^ T-cell (CTL) subsets were associated with TFR. Accordingly, “activated CTL clones” in this study should be interpreted as Vβ-skewed CD8^+^ T-cell populations detected by flow cytometry, rather than functionally validated tumor-antigen-specific CTLs. Cytotoxic functional confirmation will be required in future studies.

Specifically, patients with a sustained DMR of ≥1 year, a prolonged duration of TKI treatment of ≥7 years, and robust activation of effector and memory CTLs were associated with higher rates of TFR (Table 4). In clinical practice, it is currently unfeasible to routinely demonstrate CML stem cell-specificity of CTLs via functional assays or tetramer-based studies. Previous research has demonstrated the presence of RR-1-specific CTLs in patients with CML treated with interferon-alpha, thereby supporting the concept that autologous CTLs can mediate anti-leukemic effects [20]. The strength of our study lies in identifying an easily accessible, clinically applicable immune marker—namely, the activation status and clonality of CTLs, measurable using flow cytometry without requiring advanced molecular techniques. We acknowledge the need for further studies to definitively establish antigen specificity; however, the reproducible correlation between CTL activation and TFR acquisition supports the biological relevance of our results. Furthermore, the presence of highly activated memory CTL clones in patients who sustained TFR indicates the presence of functional anti-CML CTLs, although their precise specificity remains to be characterized. We acknowledge that we did not evaluate additional phenotype markers reflecting T-cell functional states, such as exhaustion markers (e.g., PD-1, TIM-3, LAG-3, TIGIT). Incorporation of these markers may provide deeper mechanistic insight and could further refine immunological prediction of TFR in future studies.

A further limitation was that we did not address the question of how many flow cytometric immunoassays should be performed when considering TKI discontinuation. Although we have no precise answer to this question, we believe that at least two tests performed 3 months apart are necessary before making such a treatment decision. Ideally, the immunologic tests should be performed at the same time as the MR tests every 3 months.

This study has not only identified new immune markers for the acquisition of TFR but has also provided results that offer new insights into MR depth fluctuations in TKI-treated CML patients. Among the 32 patients in our cohort who achieved TFR throughout the observation period, 12 (37.5%) showed MR depth fluctuations (Supplemental Table S3 and Figure 2). In these patients, we posit that, when the depth of the MR became shallow, indicating an increase in CML stem cells, the CTLs may have suppressed the proliferation of these cancer cells and maintained the TFR. Since TKI alone cannot eradicate CML stem cells [35], both CTLs and TKIs may be needed for CML disease control. Previous research has found a higher rate of TFR in patients with specific HLAs, suggesting the activation of CML-specific CTLs in the patient’s body [29]. Notably, HLA alleles shape antigen presentation and can influence the TCR repertoire, including preferential usage of certain TCR Vβ segments; therefore, inter-individual differences in HLA background may partly modulate the observed CTL clonal architecture and Vβ distribution. HLA typing was not mandatory in this study and was not performed in most of the patients. Future research that combines HLA typing with analyses of *TCR Vβ* genes of activated CTL clones—ideally together with functional validation—may facilitate the identification of novel CML-associated antigens. This, in turn, could lead to the development of novel therapies such as chimeric antigen receptor T-cell therapy, bispecific monoclonal antibody therapy, and adoptive TCR gene-transduced T-cell therapy for refractory CML and Philadelphia chromosome-positive acute lymphoblastic leukemia.

It would be interesting to observe the changes in the MR and TCR repertoires of the patients in our cohort who did not achieve TFR and were retreated with TKIs. A further potential direction for future research is TCR repertoire analysis of CTLs in patients treated with asciminib, an ABL myristoyl pocket inhibitor with a different mechanism of action to conventional TKIs [36]. The evaluation of possible markers of T lymphocyte exhaustion may also prove fruitful in future investigations.

In conclusion, this observational study identified candidate immunologic markers associated with TFR acquisition in patients with CP–CML treated with TKIs. These CTL-related metrics are potentially implementable in routine practice using flow cytometry, and may help bridge immunological insights with clinical decision-making; however, their clinical utility requires confirmation in independent cohorts.

## 4. Materials and Methods

### 4.1. Patients

Patients with CP–CML were eligible for inclusion if they met at least one of the following criteria.

1. Sustained DMR (≥MR4) for >1 year while continuing TKI treatment (Criteria 1; On-TKI group), or
2. Sustained DMR for >1 year after prior discontinuation of TKI treatment (Criteria 2; Off-TKI group).

Patients were required to be ≥20 years of age. This study included patients who desired to attempt TKI discontinuation, in consultation with their treating physician, after meeting eligibility criteria.

Criterion 1 was achieved by 38 patients, categorized as the On-TKI group. They discontinued TKI therapy at the enrollment. Criterion 2 was fulfilled by seven patients, classified as the Off-TKI group.

Exclusion criteria included:

1. Mental illness or psychiatric symptoms that interfere with participation or informed consent.
2. Known immunodeficiency or current immunosuppressive therapy.
3. Any condition deemed unsuitable by the treating physician.

The study registration period was 1 July 2019 to 31 March 2022. A total of 45 patients were enrolled from three Japanese institutions: Japanese Red Cross Nagasaki Genbaku Hospital, Oita Prefectural Hospital, and Chugoku Central Hospital.

### 4.2. Study Design

This was a non-randomized, non-blinded, prospective observational study approved by the Central Ethical Review Board of the Japanese Red Cross Nagasaki Genbaku Hospital (approval no. 597). The study was conducted in accordance with the principles of the 1964 Declaration of Helsinki and its later revisions.

Immunological evaluations, including MR status and tumor immunity markers, were conducted for 1 year from the time of patient enrollment and consent.

### 4.3. Analysis of Biomarkers

MR status was assessed using quantitative real-time polymerase chain reaction (qRT-PCR) for *bcr-abl1* mRNA, reported on the international scale (IS) [37].

- MR5: *bcr::abl1*^IS^ ≤ 0.001%
- MR4.5: *bcr::abl1*^IS^ ≤ 0.0032%
- MR4: *bcr::abl1*^IS^ ≤ 0.01%.
- DMR: defined as ≥ MR4.
- TFR failure was defined as loss of MR4.

Immunological parameters (T-cell subsets, NK cells, regulatory T-cells [Tregs], granulocytic [G]- and monocytic [M]-myeloid-derived suppressor cells [MDSCs], and CTL clonality) were analyzed by flow cytometry. T-cell subsets were assessed using the CYTE-STAT tetraCROME Kit (Beckman Coulter, Brea, CA, USA; catalog number 6607013). For surface immunophenotyping other than Treg analysis, erythrocytes were lysed using OptiLyse C Lysing Solution (Beckman Coulter, Brea, CA, USA; catalog number A11895). Treg analysis requiring intracellular FOXP3 staining was performed using the PerFix-nc Kit (Beckman Coulter, Brea, CA, USA; catalog number B31167). TCR clonality of CD8^+^ T-cells was assessed using the IOTest Beta Mark TCR Vβ Repertoire Kit (Beckman Coulter, Brea, CA, USA; catalog number IM3497), following the manufacturer’s protocol.

Flow cytometry acquisition and analysis:

Flow cytometric acquisition was performed on a NAVIOS EX flow cytometer (Beckman Coulter, Tokyo, Japan), and data were analyzed using Kaluza Analysis v2.2 (Beckman Coulter).

Surface immunophenotyping (T-cell subsets, NK cells, MDSCs, and TCR repertoire):

For surface staining panels, including T-cell subsets assessed using the CYTE-STAT tetraCROME Kit and the CTL, NK, MDSC, and TCR Vβ panels, whole-blood samples were incubated with the corresponding fluorochrome-conjugated monoclonal antibodies for 10 min at room temperature. Erythrocytes were lysed using OptiLyse C Lysing Solution (Beckman Coulter) according to the manufacturer’s instructions. After lysis, cells were washed once with phosphate-buffered saline and resuspended for acquisition. No fixation/permeabilization step was performed for these panels. Representative flow cytometric gating plots for T-cell subsets, NK cells, effector Tregs, G-MDSCs, and M-MDSCs are provided in Supplemental Figure S1.

Treg analysis with fixation/permeabilization:

For Treg analysis requiring intracellular FOXP3 staining, samples were processed using the high-fixation/permeabilization procedure of the PerFix-nc Kit (Beckman Coulter; catalog number B31167). Briefly, 50 µL of whole blood was added to each labeled tube, followed by 25 µL of PerFix-nc Buffer 1 (Fixative Reagent). Samples were vortexed immediately and incubated for 15 min at room temperature (18–25 °C). Subsequently, 300 µL of PerFix-nc Buffer 2 (Permeabilizing Reagent) was added, and the samples were vortexed immediately. Fluorochrome-conjugated antibodies against intracellular and relevant surface antigens were then added, followed by incubation for 15–30 min at room temperature. Finally, 3 mL of Final 1× Reagent was added. This reagent was freshly prepared on the day of use by diluting PerFix-nc Buffer 3 (Final 10× Solution) 1:10 in deionized water (1 volume of Buffer 3 with 9 volumes of water), according to the manufacturer’s instructions. Samples were then acquired on the flow cytometer. An optional final wash step described in the manufacturer’s instructions was not routinely applied.

Details of antibodies, kits, clone names, catalog numbers, fluorochromes, and manufacturers are provided in Supplemental Table S4.

Gating strategy:

For analyses of T-cell subsets and NK cells, T-cells were identified as CD3^+^ populations (e.g., CD3^+^CD4^+^ and CD3^+^CD8^+^), and NK cells were identified within CD3^−^CD16/CD56^+^ events. In contrast, CD3 was not included in the antibody panels used for CTL phenotyping and for the TCR Vβ repertoire assay (Supplemental Table S4). CTL subsets were defined within CD8^+^ lymphocytes using CD27 and CD45RA (naïve: CD8^+^CD27^+^CD45RA^+^; effector: CD8^+^CD27^−^CD45RA^+^; memory: CD8^+^CD45RA^−^). For TCR Vβ repertoire analysis, lymphocytes were gated by Forward scatter/Side scatter, followed by gating on CD8^+^ events, and Vβ family frequencies were determined using the IO Test Beta Mark Vβ Repertoire Kit (Beckman Coulter) according to manufacturer’s instructions. Because CD3 was not included in the TCR Vβ panel, minimal NK contamination cannot be completely excluded; however, NK cells do not express TCR Vβ and thus would not artifactually increase a Vβ family frequency. Naïve/effector/memory subset frequencies were calculated as percentages of total CD8^+^ cells (CD8^+^ = 100%). Vβ family frequencies were calculated within each subset gate (i.e., within effector CTLs or within memory CTLs, respectively). Representative flow cytometric gating plots for T-cell subsets, NK cells, effector Tregs, G-MDSCs, and M-MDSCs are provided in Supplemental Figure S1.

CTL subsets were defined [24] as:

- Naïve CTLs: CD8^+^CD27^+^CD45RA^+^
- Effector CTLs were defined as CD8^+^CD27^−^CD45RA^+^
- Memory CTLs were defined as CD8^+^CD27^+/−^CD45RA^−^

Clonal expansion was categorized as:

- Weakly activated clones: 5–9.9%
- Activated clones: 10–14.9%
- Highly activated CTL clones: ≥15% of a given Vβ family.

For TCR Vβ clonality, Vβ families meeting the predefined thresholds (weakly activated 5–9.9%, activated 10–14.9%, highly activated ≥ 15%) were reported; in Table 3, clonality is summarized by reporting the corresponding Vβ gene family names rather than displaying the numeric percentages.

In this study, “CTL” refers to phenotypically defined CD8^+^ T lymphocyte subsets (CD27/CD45RA-based naïve/effector/memory classification) and does not necessarily indicate confirmed cytotoxic function. According to the manufacturer’s reference data in normal specimens, the relative representation of individual TCR Vβ families varies widely across Vβ families in CD3^+^ and CD3^+^CD8^+^ T-cell subsets; therefore, the terms “weakly activated/activated/highly activated” were used as an operational descriptor of Vβ over-representation within effector/memory CTL subsets rather than as a surrogate for functional activation. Definitive clonality and antigen specificity require sequencing and/or functional assays.

For MDSC analyses, results were expressed as frequencies (ratios) relative to total nucleated cells in whole blood.

Human leukocyte antigen (HLA) typing was not mandatory; thus, CML-specific CTLs could not be directly obtained.

Assessment schedule:
  - On-TKI group: MR and immune markers assessed at 0, 1, 2, 3, 4, 5, 6, 8, 10, and 12 months after TKI discontinuation.
  - Off-TKI group: Evaluated at 0, 2, 4, 6, 8, 10, and 12 months post-enrollment.
  - All time points were flexible based on outpatient visits.

### 4.4. Endpoints

The primary endpoint was to investigate the relationship between TFR status and CD3^+^CD8^+^ CTL clonality following TKI discontinuation in patients who maintained DMR for >1 year.

### 4.5. Statistical Analysis

All statistical analyses were conducted using GraphPad Prism 9 (GraphPad Software, San Diego, CA, USA) software. The Mann–Whitney U test was used to compare continuous variables, whereas categorical data were analyzed using Fisher’s exact test. A *p*-value < 0.05 indicated statistical significance.

## Figures and Tables

**Table 1:** Clinical characteristics of the study cohort at baseline.

	On-TKI Group * (*n* = 38)	Off-TKI Group ** (*n* = 7)	*p* Value
Age, years, median (range)	57.5 (29–85)	65 (56–87)	0.058
Male/Female	25/13	5/2	>0.999
Sokal score *n* (%)			
Low	20 (53)	2 (29)	
Intermediate	10 (26)	3 (43)	0.492
High	8 (21)	1 (14)	
Missing values	0 (0)	1 (14)	
Hasford score *n* (%)			
Low	15 (39)	3 (43)	
Intermediate	19 (50)	3 (43)	0.680
High	4 (11)	0 (0)	
Missing values	0 (0)	1 (14)	
ELTS score *n* (%)			
Low	35 (92)	6 (86)	
Intermediate	3 (8)	0 (0)	n/a
High	0 (0)	0 (0)	
Missing values	0 (0)	1 (14)	
Duration of TKI treatment, years			
Median (range)	7.65 (1.5–17.4)	10.0 (3.3–18.3)	0.3568
Time since TKI discontinuation at study onset, years			
Median (range)	n/a	4.9 (1.1–7.1)	n/a

* Patients who had maintained DMR for ≥1 year with TKI treatment. ** Patients who had maintained DMR for ≥1 year after discontinuation of TKI treatment. DMR, deep molecular response; ELTS, EUTOS long-term survival; n/a, not applicable; TKI, tyrosine kinase inhibitor.

**Table 2:** Molecular response status during the observation period in all patients enrolled in the study.

	Maintenance of DMR *n* (%)	Loss of DMR *n* (%)	*p* Value
On-TKI group *n* = 38	25 (65.8%)	13 (34.2%)	
Off-TKI group *n* = 7	7 (100%)	0 (0%)	
Duration of TKI treatment (years) Median (range)	9.45 (3.3–18.3)	4.9 (1.5–11.9)	0.005

The On-TKI group included patients who had maintained DMR for ≥1 year with TKI treatment. The Off-TKI group included patients who had maintained DMR for ≥1 year after TKI discontinuation. DMR, deep molecular response; TKI, tyrosine kinase inhibitor.

**Table 3:** Proportions of CD4^+^ and CD8^+^ T cells, and CTL subsets and CTL clonality in patients who lost their DMR after participating in this study, despite > 10 years of TKI treatment.

Patient ID	LD-4	LD-7	LD-11	LD-13
Duration of TKI treatment (years)	Ima: 0.2, Dasa: 10.8 (Total: 11.0)	Ima: 4.1, Nilo: 0.01, Ima: 3.4, Dasa: 4.4 (Total: 11.91)	Ima: 10.4	Ima: 11.4
CD4 (%)	36.0	34.8	37.9	18
CD8 (%)	10.1	27.8	9.2	10.2
CD4/CD8	3.6	1.2	4.2	1.8
Naïve CTLs (%)	22.6	11.3	32.5	8.3
Effector CTLs (%)	50.2	71.8	44.9	68.3
Memory CTLs (%)	23.7	14.0	16.7	20.6
CTL clonality				
Effector CTLs				
Highly activated:	(−)	(−)	Vb9	(−)
Activated:	(−)	Vb2	(−)	Vb23
Weakly activated:	Vb8	(−)	(−)	Vb13.1
Memory CTLs				
Highly activated:	(−)	(−)	(−)	(−)
Activated:	Vb13.1, Vb2	Vb2	(−)	Vb13.6
Weakly activated:	Vb1, Vb3, Vb14, Vb22	Vb17, Vb3	Vb9	Vb1

The values for CD4^+^ and CD8^+^ T-cells, CD4^+^/CD8^+^ ratio, and naïve, effector, and memory CTL subsets represent mean values calculated from baseline to the time of DMR loss. Percentages of naïve, effector, and memory CTLs are expressed as proportions of total CD8^+^ cells (CD8^+^ T-cells = 100%). For CTL clonality, the presence of weakly activated (5–9.9%), activated (10–14.9%), or highly activated (≥15%) Vβ populations are reported by listing the corresponding *TCR Vβ* gene family names. When no such Vβ population was detected, “(−)” is shown. Percentages are not displayed. CD4, cluster of differentiation 4; CD8: cluster of differentiation 8; CTL, cytotoxic T lymphocyte; Dasa, dasatinib; DMR, deep molecular response; ID, identification; Ima, imatinib; LD, loss of DMR; Nilo, nilotinib; TCR, T-cell receptor; TKI, tyrosine kinase inhibitor; Vb: *TCR Vβ* gene segments expressed by CTL clones.

**Table 4:** Conditions required for safe discontinuation of tyrosine kinase inhibitor treatment.

**Essential Conditions:**	Tyrosine kinase inhibitor treatment duration of ≥7 years Deep molecular response maintained for ≥1 year
**Condition 1:**	CD8 > CD4 in T lymphocyte ratio
**Condition 2:**	Memory cytotoxic T lymphocytes > effector cytotoxic T lymphocytes in total cytotoxic T lymphocyte ratio
**Condition 3:**	Presence of a highly activated memory cytotoxic T lymphocyte clone
**Condition 4:**	Presence of a highly activated effector cytotoxic T lymphocyte clone
**Safety Condition 1.**	Essential conditions and at least one of conditions 1–3 are met: The probability of treatment-free remission is close to 100%
**Safety Condition 2.**	Essential conditions and condition 4 are met but none of conditions 1–3: The probability of treatment-free remission is >90%

**Table 5:** Association between candidate immunological conditions and sustained DMR after TKI discontinuation in patients treated for ≥7 years.

	TKI ≥7 Years &	TKI ≥7 Years &	*p* Value	Odds Ratio for DMR Maintenance
	Maintenance of DMR	Loss of DMR		(95% Confidence Interval)
	*n* = 21	*n* = 4		
CD8 > CD4	16	0	0.0100	Infinity
				(1.950 to infinity)
Total memory CTLs > total effector CTLs	7	0	0.2945	Infinity
				(0.3270 to infinity)
Presence of highly activated memory CTL clone	3	0	>0.9999	Infinity
				(0.1443 to infinity)
Presence of highly activated effector CTL clone	10	1	0.6043	2.727
				(0.3424 to 38.50)

Univariate comparisons of four candidate immunological conditions between patients with sustained DMR (*n* = 21) and DMR loss (*n* = 4) among patients treated with TKIs ≥7 years are shown. Values indicate the number of patients meeting each condition. *p* values were calculated using Fisher’s exact test. Odds ratios (ORs) and 95% confidence intervals are presented with DMR maintenance as the outcome (maintenance vs. loss). Because of zero cells in some comparisons, ORs were infinite in those instances. DMR, deep molecular response; TKI, tyrosine kinase inhibitor; CTL, cytotoxic T lymphocyte.

## Data Availability

The data supporting the findings of this study are available from the corresponding author upon reasonable request. These data are not publicly available due to privacy or ethical restrictions.

## Supplementary Materials

The supporting information can be downloaded at: https://www.mdpi.com/article/10.3390/ijms27062713/s1.
