# Supplementary material for: Activated Memory Cytotoxic T-Lymphocytes and T-Cell Receptor Vβ Clonality Predict Treatment-Free Remission After Tyrosine Kinase Inhibitor Discontinuation in Chronic-Phase Chronic Myeloid Leukemia: A 1-Year Prospective Immuno-Monitoring Study
Source: Int J Mol Sci. 2026 Mar 16;27(6):2713. doi: 10.3390/ijms27062713 (PMC13026259; doi:10.3390/ijms27062713)
Supplement: Supplementary file 1 [file ijms-27-02713-s001.zip › Supplemental Figures S1 & S2_R3.pdf]

(A)

MD-21

CD3⁺ T-cell: 58.1%
CD3⁺CD4⁺ T-cell: 28.8%
CD3⁺CD8⁺ T-cell: 26.7%

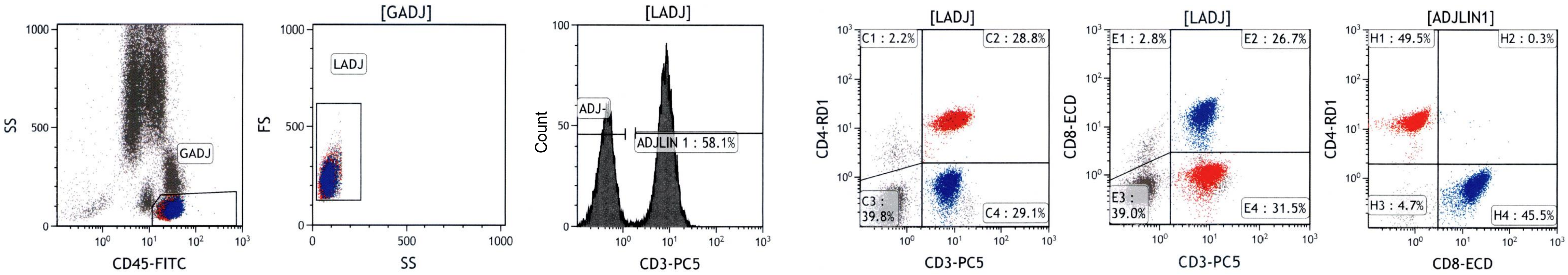

LD-3

CD3⁺ T-cell: 52.7%
CD3⁺CD4⁺ T-cell: 20.1%
CD3⁺CD8⁺ T-cell: 32.2%

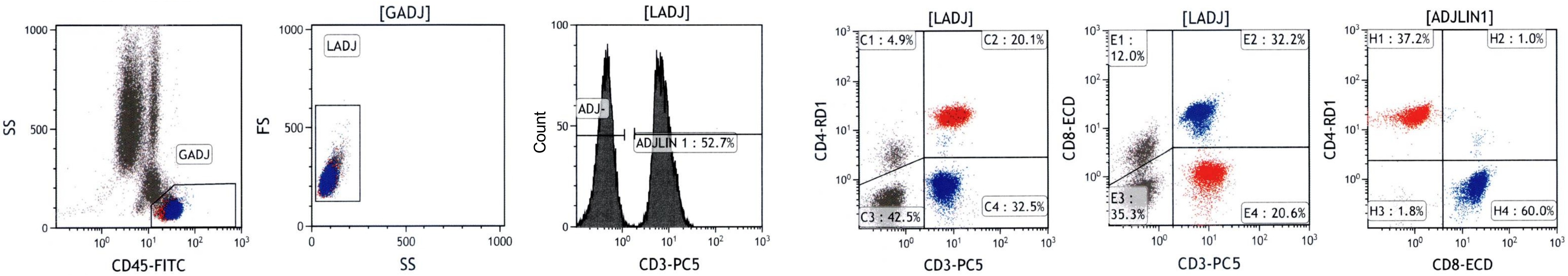

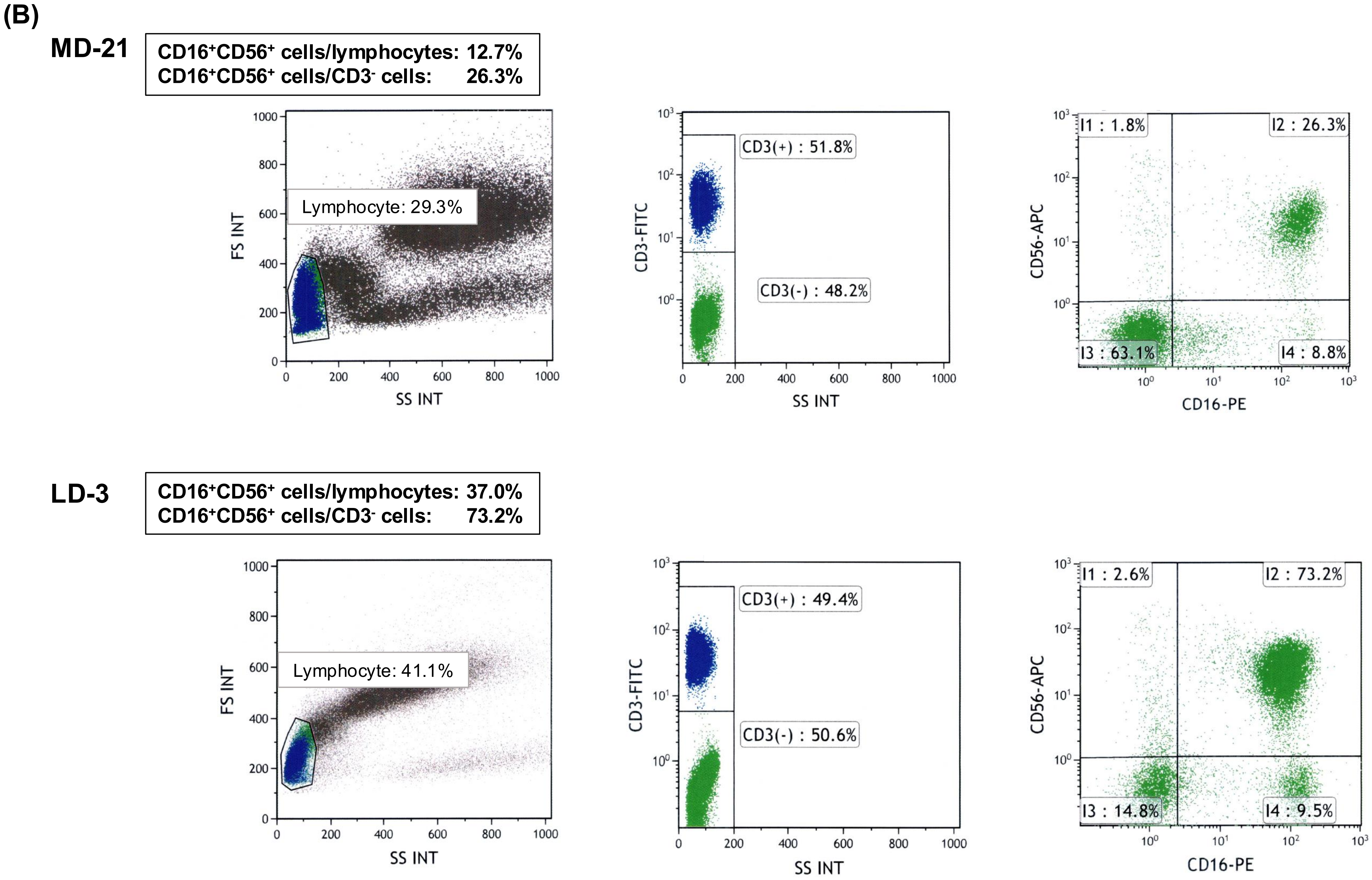
(B)
MD-21
CD16+CD56+ cells/lymphocytes: 12.7%
CD16+CD56+ cells/CD3- cells: 26.3%
Lymphocyte: 29.3%
FS INT
SS INT
CD3(+) : 51.8%
CD3(-) : 48.2%
CD3-FITC
I1 : 1.8%
I2 : 26.3%
I3 : 63.1%
I4 : 8.8%
CD56-APC
CD16-PE
LD-3
CD16+CD56+ cells/lymphocytes: 37.0%
CD16+CD56+ cells/CD3- cells: 73.2%
Lymphocyte: 41.1%
CD3(+) : 49.4%
CD3(-) : 50.6%
I1 : 2.6%
I2 : 73.2%
I3 : 14.8%
I4 : 9.5%

(C)

MD-21

Non-Treg: 1.7%
Naïve Treg: 0.2%
Effector Treg: 0.0%

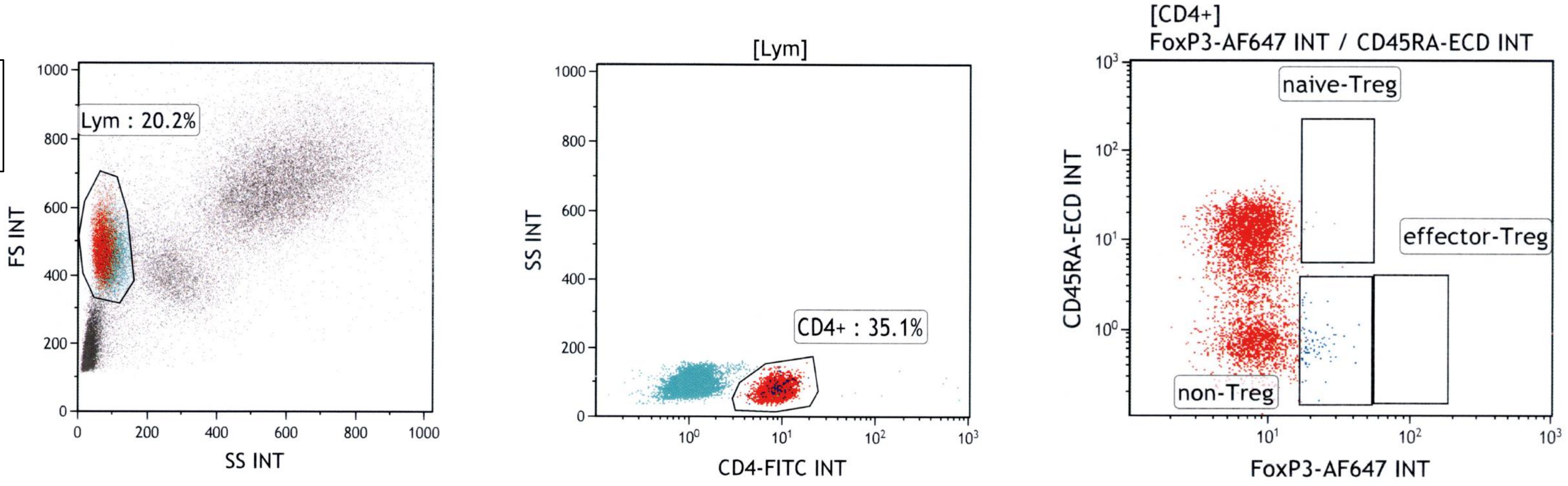

LD-3

Non-Treg: 5.2%
Naïve Treg: 1.3%
Effector Treg: 1.2%

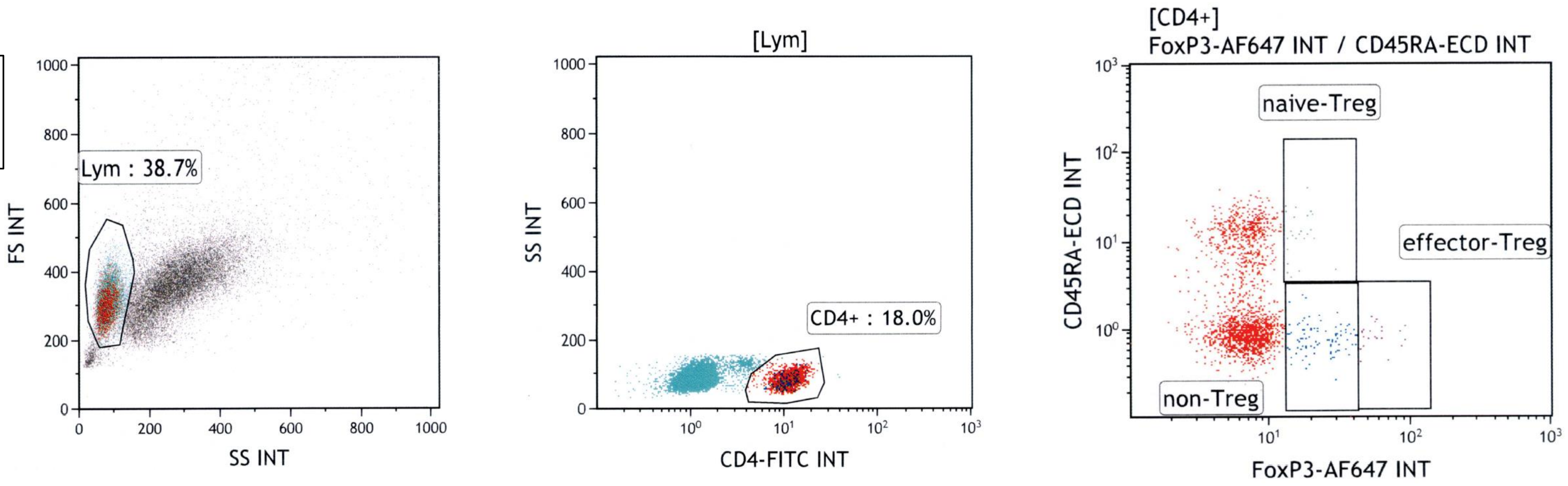

(D)

MD-21

G-MDSC: 34.0%
M-MDSC: 0.2%

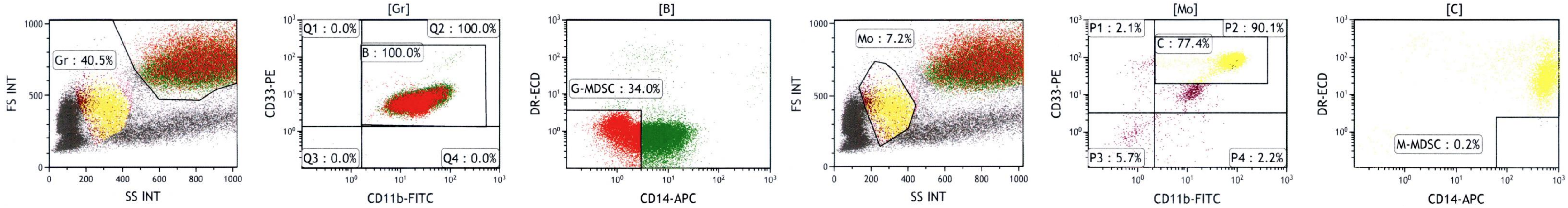

LD-3

G-MDSC: 35.1%
M-MDSC: 3.3%

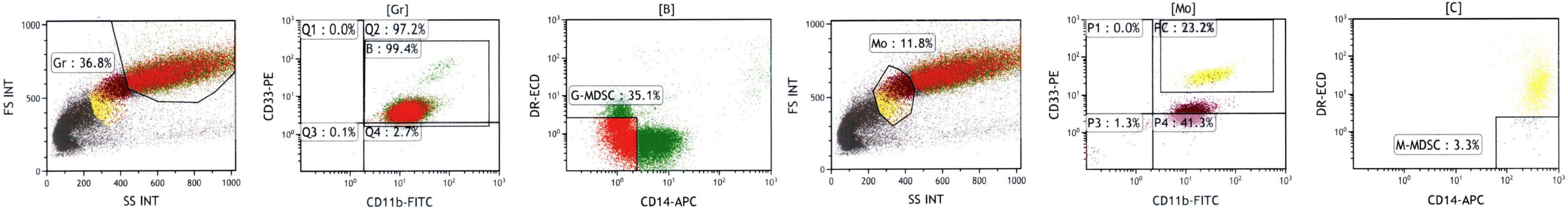

Supplemental Figure S1. Representative flow cytometric gating plots for T-cell subsets, NK cells, effector Tregs, G-MDSCs, and M-MDSCs.

Legend:

(A) T-cell subset plots from two representative patients displayed side by side: MD-21, who maintained TFR for 12 months after TKI discontinuation, and LD-3, who lost DMR at 6 months after discontinuation. For each patient, the sequential gating strategy is shown as CD45/SSC gating, identification of $CD3^{+}$ T-cells, and subdivision into $CD3^{+}CD4^{+}$ T-cells and $CD3^{+}CD8^{+}$ T-cells.

(B) NK cell plots from MD-21 and LD-3 displayed side by side. For each patient, the sequential gating strategy is shown as FSC/SSC lymphocyte gating, identification of $CD3^{-}$ cells, and subdivision into $CD16^{+}CD56^{+}$ NK cells.

(C) Effector Treg plots from MD-21 and LD-3 displayed side by side. For each patient, the sequential gating strategy is shown as FSC/SSC lymphocyte gating, identification of $CD4^{+}$ cells, and subdivision into non-Tregs, naïve Tregs, and effector Tregs.

(D) G-MDSC and M-MDSC plots from MD-21 and LD-3 displayed side by side. For each patient, the sequential gating strategy for G-MDSCs is shown as FSC/SSC granulocyte gating, identification of $CD11b^{+}CD33^{+}$ cells, and subdivision into G-MDSCs ($CD14^{-}$HLA-DR^{-}). The sequential gating strategy for M-MDSCs is shown as FSC/SSC monocyte gating, identification of $CD11b^{+}CD33^{+}$ cells, and subdivision into M-MDSCs ($CD14^{+}$HLA-DR^{-}).

Abbreviations:

AF647, Alexa Fluor 647; APC, allophycocyanin; ECD, phycoerythrin-Texas Red; FITC, fluorescein isothiocyanate; FS, forward scatter; INT, intensity; PC5, phycoerythrin-Cy5; PE, phycoerythrin; SS, side scatter

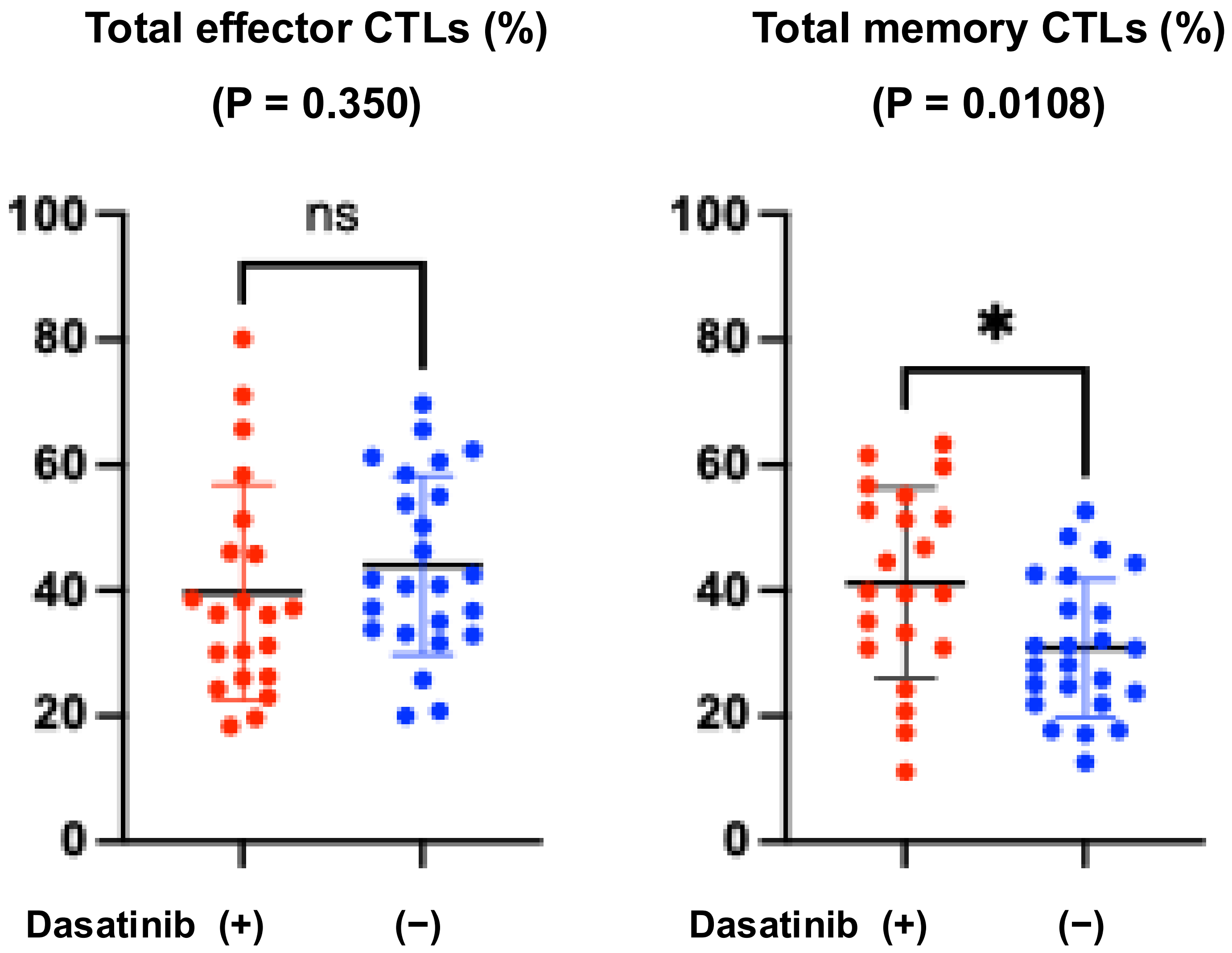
Total effector CTLs (%)
(P = 0.350)
Total memory CTLs (%)
(P = 0.0108)
ns
*
100
80
60
40
20
0
Dasatinib (+)
(−)

Supplemental Figure S2. Association between prior dasatinib exposure and effector and memory CTL proportions during the study period

Legend:

Scatter plots compare the proportions of total effector (left) and total memory CTLs (right) between patients with prior dasatinib exposure [Dasatinib (+), n = 21] and those without prior dasatinib exposure [Dasatinib (-), n = 24]. For each patient, values represent the mean percentage across the observation period. Each dot indicates an individual patient; horizontal bars indicate mean ± standard deviation. P values were calculated using a two-tailed unpaired t-test (effector CTLs: $P = 0.350$; memory CTLs: $P = 0.0108$). Statistical significance ($P < 0.05$) is indicated by an asterisk; ns, not significant.
