# Supplementary material for: Activated Memory Cytotoxic T-Lymphocytes and T-Cell Receptor Vβ Clonality Predict Treatment-Free Remission After Tyrosine Kinase Inhibitor Discontinuation in Chronic-Phase Chronic Myeloid Leukemia: A 1-Year Prospective Immuno-Monitoring Study
Source: Int J Mol Sci. 2026 Mar 16;27(6):2713. doi: 10.3390/ijms27062713 (PMC13026259; doi:10.3390/ijms27062713)
Supplement: Supplementary file 1 [file ijms-27-02713-s001.zip › Supplemental Table S1-4_R3.pdf]

Supplemental Table S1. Proportions of $CD4^+$ and $CD8^+$ T cells, and CTL subsets, and CTL clonality in patients treated with TKIs <10 years who lost DMR after study enrollment

Patient ID	LD-1	LD-2	LD-3	LD-5	LD-6
**Duration of TKI treatment (years)**	Dasa: 4.0	Dasa: 1.7	Nilo: 0.4<br>Dasa: 4.3<br>(Total: 4.7)	Dasa: 4.7	Nilo: 0.2<br>Dasa: 4.0<br>(Total: 4.2)
**CD4 (%)**	25.0	50.7	23.0	19.8	32.5
**CD8 (%)**	31.5	19.3	30.4	19.7	28.5
**CD4/CD8**	0.79	2.62	0.77	1.03	1.15
**Naïve CTLs (%)**	23.2	22.6	9.2	10.2	9.0
**Effector CTLs (%)**	37.6	46.5	80.5	61.6	40.5
**Memory CTLs (%)**	32.1	24.1	6.9	20.2	44.2
**CTL clonality**					
**Effector CTLs**					
**Highly activated:**	(−)	Vb1	Vb13.1	(−)	(−)
**Activated:**	(−)	(−)	(−)	Vb5.1, Vb14	(−)
**Weakly activated:**	Vb3, Vb14	Vb3, Vb9, Vb13.2	Vb13.2	(−)	Vb14
**Memory CTLs**					

**Highly activated:**	(−)	(−)	(−)	(−)	(−)
**Activated:**	Vb20	Vb1, Vb22	Vb13.2	(−)	(−)
**Weakly activated:**	Vb14, Vb13.2	Vb8, Vb13.2	Vb7.1, Vb3, Vb2, Vb13.1	Vb17, Vb14	Vb17, Vb14

**Patient ID**	**LD-8**	**LD-9**	**LD-10**	**LD-12**
**Duration of TKI treatment (years)**	Dasa: 6.1	Dasa: 1.5	Dasa: 2.3	Nilo: 5.1
**CD4** (%)	35.0	15.5	23.4	26.4
**CD8** (%)	16.7	33.9	14.0	27.8
**CD4/CD8**	2.12	0.46	1.68	0.96
**Naïve CTLs** (%)	11.9	12.3	20.2	47.1
**Effector CTLs** (%)	31.0	30.3	34.5	20.6
**Memory CTLs** (%)	52.0	50.4	38.8	27.7
**CTL clonality**				
**Effector CTLs**				
**Highly activated:**	(−)	Vb21.3	(−)	Vb2
**Activated:**	(−)	Vb3	(−)	(−)
**Weakly activated:**	(−)	Vb16	Vb21.3, Vb4, Vb5.3, Vb1	Vb8

**Memory CTLs**				
**Highly activated:**	(−)	Vb21.3	(−)	(−)
**Activated:**	Vb17	(−)	(−)	Vb2, Vb4
**Weakly activated:**	(−)	Vb3	Vb7.1, Vb2, Vb5.3	(−)

The values for $CD4^+$ and $CD8^+$ T cells, $CD4^+/CD8^+$ ratio, and naïve, effector, and memory CTL subsets represent mean values calculated from baseline to the time of DMR loss.

CD4, cluster of differentiation 4; CD8, cluster of differentiation 8; CTL, cytotoxic T lymphocyte; Dasa, dasatinib; DMR, deep molecular response; ID, identification; LD, loss of DMR; Nilo, nilotinib; TCR, T-cell receptor; TKI, tyrosine kinase inhibitor; Vb, TCR Vβ gene segments expressed by CTL clones

Supplemental Table S2. Proportions of $CD4^+$, $CD8^+$, and CTL subsets and CTL clonality in patients treated with TKI for ≥7 years who maintained TFR throughout the study period

Patient ID	MD-7	MD-19	MD-8	MD-18
**Duration of TKI treatment (years)**	Nilo: 0.5<br>Dasa: 4.0<br>(Total: 7.0)	Nilo: 4.5<br>Bosu: 2.5<br>(Total: 7.0)	Ima: 7.1	Nilo: 7.4
**CD4** (%)	21.2	32.0	15.2	43.6
**CD8** (%)	36.5	37.2	7.99	16.4
**CD4/CD8**	0.61	0.87	1.89	2.66
**Naïve CTLs** (%)	18.1	12.2	10.6	37.3
**Effector CTLs** (%)	30.5	70.2	46.1	41.4
**Memory CTLs** (%)	44.4	13.7	38.7	19.3
**CTL clonality**				
**Effector CTLs**				
**Highly activated:**	Vb11	Vb2	(−)	Vb13.1
**Activated:**	Vb14	(−)	(−)	(−)
**Weakly activated:**	Vb13.2	(−)	(−)	Vb9
**Memory CTLs**				
**Highly activated:**	(−)	(−)	(−)	(−)

**Activated:**	(−)	(−)	(−)	(−)
**Weakly activated:**	Vb13.2, Vb2, Vb1, Vb22	Vb3, Vb9	Vb22, Vb13.1	Vb14, Vb2, Vb7.1

**Patient ID**	**MD-17**	**MD-3**	**MD-16**	**MD-6**
**Duration of TKI treatment (years)**	Nilo: 7.5	Nilo: 0.02, Dasa: 0.2<br>Nilo: 1.6, Dasa: 5.7<br>(Total: 7.52)	Nilo: 7.7	Nilo: 5.9<br>Dasa: 2.6<br>(Total: 8.5)
**CD4** (%)	39.0	31.7	31.2	29.5
**CD8** (%)	41.5	25.0	30.6	15.6
**CD4/CD8**	0.94	1.28	1.11	1.90
**Naïve CTLs** (%)	17.9	14.6	18.9	44.0
**Effector CTLs** (%)	33.5	27.1	56.5	32.4
**Memory CTLs** (%)	40.5	50.3	20.5	19.0
**CTL clonality**				
**Effector CTLs**				
**Highly activated:**	Vb1	(−)		(−)
**Activated:**	(−)	Vb1, Vb13.1	Vb13.1	(−)
**Weakly activated:**	Vb7.1, Vb22	Vb22	(−)	(−)
			Vb20, Vb1, Vb7.1	

**Memory CTLs**				
**Highly activated:**	Vb17	(−)		(−)
**Activated:**	(−)	Vb3	(−)	(−)
**Weakly activated:**	Vb7.1, Vb1	Vb12	(−)	Vb2, Vb17, Vb5.1
			Vb3, Vb8, Vb2, Vb7.1, Vb13.2	

Patient ID	MD-20	MD-25	MD-21	MD-5
**Duration of TKI treatment (years)**	Ima: 0.02, Dasa: 2.1,<br>Nilo: 3.0, Bosu: 0.1,<br>Nilo: 4.1<br>(Total: 9.32)	Ima: 9.5	Nilo: 9.6	Ima: 1.6<br>Nilo: 2.0<br>Dasa: 6.3<br>(Total: 9.9)
**CD4** (%)	37.8	23.4	28.4	34.3
**CD8** (%)	5.90	27.6	25.3	19.9
**CD4/CD8**	6.41	0.87	1.12	1.73
**Naïve CTLs** (%)	11.8	34.3	41.6	15.9
**Effector CTLs** (%)	70.0	37.3	33.5	33.0
**Memory CTLs** (%)	15.9	22.8	17.8	46.1
**CTL clonality**				
**Effector CTLs**				

**Highly activated:**	(−)	Vb7.1, Vb14	(−)	Vb17
**Activated:**	(−)	(−)	Vb13.1	(−)
**Weakly activated:**	(−)	(−)	Vb14	Vb22
**Memory CTLs**				
**Highly activated:**	(−)	(−)	(−)	(−)
**Activated:**	Vb2	(−)	(−)	(−)
**Weakly activated:**	(−)	Vb2, Vb7.1	Vb13.1, Vb5.3, Vb21.3, Vb2	Vb22, Vb1

**Patient ID**	**MD-12**	**MD-15**	**MD-13**	**MD-22**
**Duration of TKI treatment (years)**	Ima: 10.3	Ima: 11.3	Ima: 12.6	Ima: 13.4
**CD4** (%)	31.6	36.5	23.2	45.5
**CD8** (%)	28.0	23.2	14.2	16.4
**CD4/CD8**	1.13	1.73	1.63	2.79
**Naïve CTLs** (%)	21.3	21.2	38.0	22.1
**Effector CTLs** (%)	42.1	60.9	40.9	35.9
**Memory CTLs** (%)	30.9	13.2	16.7	37.8
**CTL clonality**				

**Effector CTLs**				
**Highly activated:**	Vb1	(−)	(−)	Vb14
**Activated:**	Vb9	Vb9, Vb14	(−)	Vb3
**Weakly activated:**	Vb17	(−)	Vb1	Vb5.1
**Memory CTLs**				
**Highly activated:**	(−)	(−)	(−)	(−)
**Activated:**	(−)	Vb2	(−)	(−)
**Weakly activated:**	Vb2, Vb17, Vb22	Vb3	Vb3, Vb2, Vb17, Vb1	Vb1, Vb7.1, Vb3

**Patient ID**	**MD-23**	**MD-10**	**MD-9**	**MD-11**	**MD-14**
**Duration of TKI treatment (years)**	Ima: 13.5	Ima: 14.3	Ima: 14.4	Ima: 16.0	Ima: 17.4
**CD4** (%)	37.3	29.0	32.8	19.3	29.0
**CD8** (%)	9.7	13.0	24.5	21.3	23.3
**CD4/CD8**	3.82	2.25	1.35	0.91	1.26
**Naïve CTLs** (%)	41.4	20.1	12.7	3.26	19.0
**Effector CTLs** (%)	26.2	36.4	56.4	51.0	66.0
**Memory CTLs** (%)	28.8	34.6	25.3	36.6	12.5

**CTL clonality**					
**Effector CTLs**					
**Highly activated:**	(−)	(−)	Vb3, Vb14	Vb2	Vb17
**Activated:**	Vb5.3	(−)	(−)	Vb13.1, Vb20	(−)
**Weakly activated:**	Vb14, Vb3	(−)	(−)	(−)	(−)
**Memory CTLs**					
**Highly activated:**	(−)	(−)	(−)	(−)	(−)
**Activated:**	Vb1, Vb13.2	Vb2	(−)	(−)	(−)
**Weakly activated:**	Vb8, Vb2	Vb17, Vb13.1, Vb1	Vb3, Vb14, Vb2	Vb2	Vb2, Vb14, Vb3, Vb7.1

The values for $CD4^+$, $CD8^+$ T cells, $CD4^+/CD8^+$ ratio, and naïve, effector, and memory CTL subsets represent mean values calculated over the observation period.

Bosu, bosutinib; CD4, cluster of differentiation 4; CD8, cluster of differentiation 8; CTL, cytotoxic T lymphocyte; Dasa, dasatinib; DMR, deep molecular response; ID, identification; Ima, imatinib; MD, maintenance of DMR; Nilo, nilotinib; TCR, T-cell receptor; TFR, treatment-free remission; TKI, tyrosine kinase inhibitor; Vb: TCR Vβ gene segments expressed by CTL clones

Supplemental Table S3. CTL clonality and proportions of CD4$^+$, CD8$^+$, and CTL subsets in patients in the Off-TKI group

Patient ID	Off-TKI-3	Off-TKI-2	Off-TKI-1	Off-TKI-6
**Duration of TKI treatment (years)**	Nilo: 3.3	Ima: 1.2<br>Dasa: 4.0<br>(Total: 5.2)	Ima: 3.4<br>Dasa: 3.8<br>(Total: 7.2)	Ima: 8.2<br>Nilo: 1.8<br>(Total: 10.0)
**Years since TKI discontinuation at study entry (years)**	2.1	4.9	5.3	6.3
**CD4** (%)	36.4	22.5	29.5	37.0
**CD8** (%)	17.4	22.9	15.4	17.8
**CD4/CD8**	1.85	0.98	1.94	2.09
**Naïve CTLs** (%)	15.3	41.8	5.8	13.0
**Effector CTLs** (%)	47.7	19.3	49.3	23.2
**Memory CTLs** (%)	33.5	33.5	41.9	57.6
**CTL clonality**				
**Effector CTLs**				
**Highly activated:**	Vb3	(−)	(−)	(−)
**Activated:**	(−)	(−)	(−)	Vb13.6
**Weakly activated:**	Vb17	Vb17, Vb2	(−)	(−)

**Memory CTLs**				
**Highly activated:**	Vb3	(−)	Vb3	Vb11
**Activated:**	(−)	(−)	(−)	(−)
**Weakly activated:**	Vb17	Vb7.1, Vb17	Vb1	Vb2, Vb7.1

Patient ID	Off-TKI-7	Off-TKI-4	Off-TKI-5
**Duration of TKI treatment (years)**	Ima: 11.1	Ima: 15.8	Ima: 18.3
**Years since TKI discontinuation at study entry (years)**	7.1	1.1	1.3
**CD4** (%)	25.0	40.2	35.0
**CD8** (%)	14.3	16.7	12.1
**CD4/CD8**	1.74	2.49	2.92
**Naïve CTLs** (%)	27.7	15.4	15.7
**Effector CTLs** (%)	26.4	62.4	65.1
**Memory CTLs** (%)	41.9	19.1	17.0
**CTL clonality**			
**Effector CTLs**			
**Highly activated:**	(−)	Vb9, Vb3, Vb13.2, Vb20	Vb17

**Activated:**	(−)	Vb21.3	(−)
**Weakly activated:**	(−)	(−)	Vb23
**Memory CTLs**			
**Highly activated:**	Vb2	(−)	(−)
**Activated:**	(−)	Vb3, Vb20	Vb8
**Weakly activated:**	Vb13.2	Vb7.1, Vb9, Vb13.2, Vb4, Vb2, Vb22	Vb1, Vb23, Vb17

The Off-TKI group consisted of patients who had maintained DMR for >1 year after TKI discontinuation. The values for $CD4^+$, $CD8^+$ T cells, and $CD4^+/CD8^+$ and naïve, effector, and memory CTL subsets represent mean values calculated over the observation period.

CD4, cluster of differentiation 4; CD8, cluster of differentiation 8; CTL, cytotoxic T lymphocyte; Dasa, dasatinib; DMR, deep molecular response; ID, identification; Ima, imatinib; Nilo, nilotinib; TCR, T-cell receptor; TKI, tyrosine kinase inhibitor; Vb, TCR Vβ gene segments expressed by CTL clones

Supplemental Table S4. Flow cytometry antibody panel

Assay	Marker/Reagent	Fluorochrome	Clone name	Catalog number	Manufacturer
T cell subsets	CYTE-STAT tetra CROME kit	–	–	6607013	Beckman Coulter
CTL subsets	CD8	APC	B9.B11	IM2469	Beckman Coulter
	CD27	PC7	1A4CC27	A54823	Beckman Coulter
	CD57	FITC	NC1	IM0466U	Beckman Coulter
NK cells	CD3	FITC	UCHT1	A07746	Beckman Coulter
	CD16	PE	3G8	A07766	Beckman Coulter
	CD56	APC	N901 (NKH-1)	A07788	Beckman Coulter
MDSCs	CD11b	FITC	Bear1	IM0530	Beckman Coulter
	CD14	APC	RMO52	IM2580	Beckman Coulter
	CD33	PE	D3HL60.251	A07775	Beckman Coulter
	HLA-DR	ECD	Immu-357	IM3636	Beckman Coulter
Tregs	CD4	FITC	13B8.2	A07750	Beckman Coulter
	CD45RA	ECD	2H4LD11LDB9 (2H4)	IM2711U	Beckman Coulter
	FOXP3	AF647	259D	B30650	Beckman Coulter
TCR repertoire	CD8	APC	B9.B11	IM2469	Beckman Coulter
	CD27	PC7	1A4CD27	A54823	Beckman Coulter
	CD45RA	ECD	2H4LD11LDB9 (2H4)	IM2711U	Beckman Coulter
	IOTest Beta Mark TCR Vβ Repertoire Kit	–	–	IM3497	Beckman Coulter

All antibodies, CYTE-STAT tetraCROME Kit, and IOTest Beta Mark TCR Repertoire Kit were purchased from Beckman Coulter, Brea, CA, USA.

CTL, cytotoxic T-lymphocyte;

NK, natural killer;

MDSCs, myeloid-derived suppressor cells;

Tregs, regulatory T cells;

TCR, T-cell receptor;

CD, cluster of differentiation;

APC, allophycocyanin;

PC7, phycoerythrin-Cy7;

FITC, fluorescein isothiocyanate;

PE, phycoerythrin;

ECD, phycoerythrin-Texas Red;

AF647, Alexa Fluor 647.
